# Incidence and risk factors for early and late reoperation following lumbar fusion surgery

**DOI:** 10.1186/s13018-022-03273-4

**Published:** 2022-08-12

**Authors:** Shuai-Kang Wang, Peng Wang, Xiang-Yu Li, Chao Kong, Jia-Yin Niu, Shi-Bao Lu

**Affiliations:** 1 Department of Orthopedics, Xuanwu Hospital, Capital Medical University, No. 45 Changchun Street, Xicheng District, Beijing, 10053 China; 2 National Clinical Research Center for Geriatric Diseases, Beijing, 10053 China; 3 Capital Med Univ, Ctr Heart, Beijing Chaoyang Hosp, Beijing, 100020 China

**Keywords:** Reoperation, Lumbar fusion, Complications, Risk factors

## Abstract

**Study design:**

Retrospective cohort study.

**Purpose:**

The aim of our study was to determine the rates and indications of reoperations following primary lumbar fusion, as well as the independent risk factors for early and late reoperation.

**Methods:**

We retrospectively reviewed patients who underwent lumbar fusion surgery between January 2017 and March 2020. All patients were followed up for more than 2 years. Characteristics, laboratory tests, primary diagnosis and surgery-related variables were compared among the early reoperation (< 3 months), the late reoperation (> 3 months) and the non-reoperation groups. Multivariable logistic regression analysis was used to identify independent risk factors for early and late reoperations.

**Results:**

Of 821 patients included in our studies, 34 patients underwent early reoperation, and 36 patients underwent late reoperation. The cumulative reoperation rate was about 4.1% (95% CI 3.8–4.5%) at 3 months, 6.2% (95% CI 5.9–6.5%) at 1 year and 8.2% (95% CI 8.0–8.5%) at 3 years. Multivariable analysis indicated that osteoporosis (odds ratio [OR] 3.6, 95% CI 1.2–10.5, *p* = 0.02) and diabetes (OR 2.1, 95% CI 1.1–4.5, *p* = 0.04) were independently associated with early reoperation and multilevel fusion (OR 2.4, 95% CI 1.1–5.4, *p* = 0.03) was independently associated with late reoperation.

**Conclusions:**

The most common reasons for early reoperation and late operation were surgical site infection and adjacent segment diseases, respectively. Osteoporosis and diabetes were independent risk factors for early reoperation, and multilevel fusion was independent risk factor for late reoperation. Surgeons should pay more attention to these patients, and future studies should consider the effects of follow-up periods on results.

## Introduction

Despite advances in analgesic and neurotrophic agents, lumbar spinal fusion surgeries performed for lumbar degenerative diseases have increased in recent years [1–3]. Compared to conservative therapy, depression plus fusion is more efficient for patients with severe nerve compression syndrome or vertebral instability. Additionally, the stability of vertebrae might be reduced after laminectomy; further posterior fusion would be needed to avoid unnecessary reoperation [4]. In a retrospective study of a Korean nationwide database, Kim et al. [2] reported a 3.5-fold increase in fusion surgery for herniated intervertebral disk disease from 2003 to 2008. A review of the Healthcare Cost and Utilization Project Nationwide Inpatient Sample in the USA found that the annual number of spinal fusion discharges increased 2.4-fold (from 174,223 to 413,171) [5].

Unplanned reoperations after lumbar fusion are defined as any indications resulting in an unplanned return to the operating room. Previous studies demonstrated the associations between reoperation and worse outcomes, including higher rates of complications and lower satisfaction [6, 7]. In a retrospective cohort study of 5022 patients, Weir et al. [8] found that mean costs in the 2 years following reoperation were £1889 higher than patients who did not undergo repeat surgery over an equivalent follow-up period. Lumbar fusion surgery is a standard method for treating lumbar degenerative diseases. Given the increase in lumbar fusion and reoperations, more efforts are needed to reduce the reoperation rate.

Previously, most studies collected data from the health insurance system based on the International Classification of Disease coding, and the specific reasons and timing of lumbar reoperations cannot be determined. There might be significant differences in indications and surgical methods for operation and reoperation across hospitals; however, few retrospective studies of the database reported the consistent standards of surgical methods. Moreover, given the differences in reasons for reoperation at various time points after fusion surgery [9], the risk factors for early and late reoperation differed. Therefore, this study aimed to determine the rates and indications of reoperations following primary fusion surgery in a prospectively collected cohort from a single teaching hospital. Furthermore, we sought to identify the independent risk factors for early and late reoperation.

## Materials and methods

This was a single-center retrospective analysis of a prospectively collected database. The institutional ethics review committee of our hospital approved the study. Surgical treatment was recommended for patients with lower back and radicular pain when conservative treatments are ineffective for more than 6 months. Before surgery, magnetic resonance imaging (MRI) and computed tomography of the spine would be used to determine the operating segments. All patients undergoing elective surgery at our center receive preoperative optimization of internal diseases (e.g., perioperative blood glucose and blood pressure control). Based on previous studies, we designed an anti-osteoporosis regimen for osteoporosis patients [10–12]. For those patients, intravenous zoledronic acid 5 mg was given on day 7 after surgery and oral calcium and vitamin D were taken daily from the initiation of therapy until the last follow-up date.

We reviewed consecutive patients who underwent elective primary transforaminal lumbar interbody fusion (TLIF) surgery from January 2017 to March 2020 for lumbar spondylolisthesis, lumbar spinal stenosis and severe degenerative disk disease. Two experienced spinal orthopedic specialists determined the surgical methods for the initial operation and reoperation based on clinical symptoms, radiographs and MRI images, and the same senior surgical team performed all operations.

### Surgical technique

Under general anesthesia, the patient was placed on the operating table in a prone position. A posterior midline incision was made for all patients. The specific surgical approach (open-Wiltse or traditional approach) was chosen based on the planned decompression range. The vertebral pedicle screws of surgical segments were implanted according to preoperative radiography and intraoperative fluoroscopy. The nerve roots were decompressed by hemilaminectomy or laminectomy according to the preoperative lumbar symptoms, radicular symptoms and MRI. After removing the intervertebral disk, the bone graft and the cage filled with bone graft were placed in the intervertebral space. At last, the remaining part of autogenous bone grafts from the decompression laminectomy was placed in the bone bed. Once the position and direction of implants were satisfactory, the wound was flushed, the drainage tube was placed, and the incision was sutured layer by layer.

### Inclusion criteria and exclusion criteria

All patients were followed up for more than 1 year after initial lumbar fusion surgery; the last follow-up date was April 1, 2022. Inclusion criteria for this study were as follows: (1) age older than 18 years, (2) patients underwent elective primary TLIF surgery due to the failure of conservative treatment and (3) surgical segments less than six. Then, patients diagnosed with spinal fracture, any spinal infection or any malignancy were excluded; moreover, we excluded patients with follow-up times less than 2 years for death or unavailable telephone number.

### Data collection

We extracted demographic variables for each included patient, including age, gender, weight, body mass index (BMI), comorbidities, bone mineral density (according to the World Health Organization criteria, osteoporosis was diagnosed when T-score ≤  − 2.5), surgical history, primary diagnosis and symptom duration. Preoperative laboratory examination data included albumin, prealbumin and hemoglobin level. The data relating to the initial operation included the American Society of Anesthesiologists level (ASA), fused levels, operative time, estimated blood loss (EBL) and order of operation. The timing and causes of reoperation were also recorded in the database. The intensity of pain was evaluated using a visual analog scale (VAS) from preoperatively to just before reoperation and at the final follow-up point. Based on the causes of reoperations, early indications (within 3 months after surgery) for reoperations included early instrumentation failures, residual stenosis and acute postoperative complications which often included surgical site infection (SSI), cerebrospinal fluid leakage, hematoma, late indications (3-month or more after surgery) for reoperation included late instrumentation failures, nonunion, late complications pain recurrence and symptomatic adjacent segment diseases (ASD). Late indications including pseudoarthrosis, nonunion and ASD are often diagnosed based on MRI, X-ray and persistent low back pain unresponsive to conservative treatment.

### Statistical analysis

All statistical analyses were performed using the SPSS software (SPSS, version 22.0, Inc., Chicago, IL, USA). The cumulative reoperation rate was estimated using the Kaplan–Meier cumulative survival function, and Kaplan–Meier curves visualized the time to reoperation. Continuous variables are expressed as the mean and standard deviation; categorical variables are expressed as frequencies with percentages. Continuous variables were analyzed using the two-tailed Student’s *t* test for normally distributed variables and the Mann–Whitney *U* test for non-normally distributed variables. Categorical variables were analyzed using the Fisher’s exact or chi-square tests. Variables with a *p* value < 0.1 in univariate analyses were further subjected to multivariate analyses. Multivariable logistic regression analysis was used to identify independent risk factors for early and late reoperations. A *p* value of 0.05 was considered significant.

## Results

**Fig. 1:**
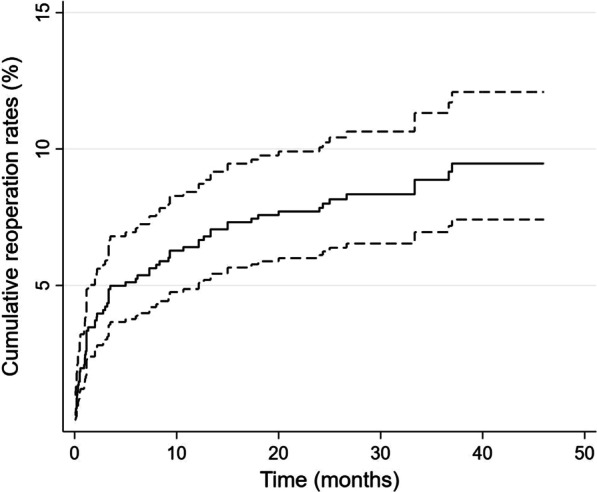
Cumulative rate of reoperation after lumbar fusion surgery

**Fig. 2:**
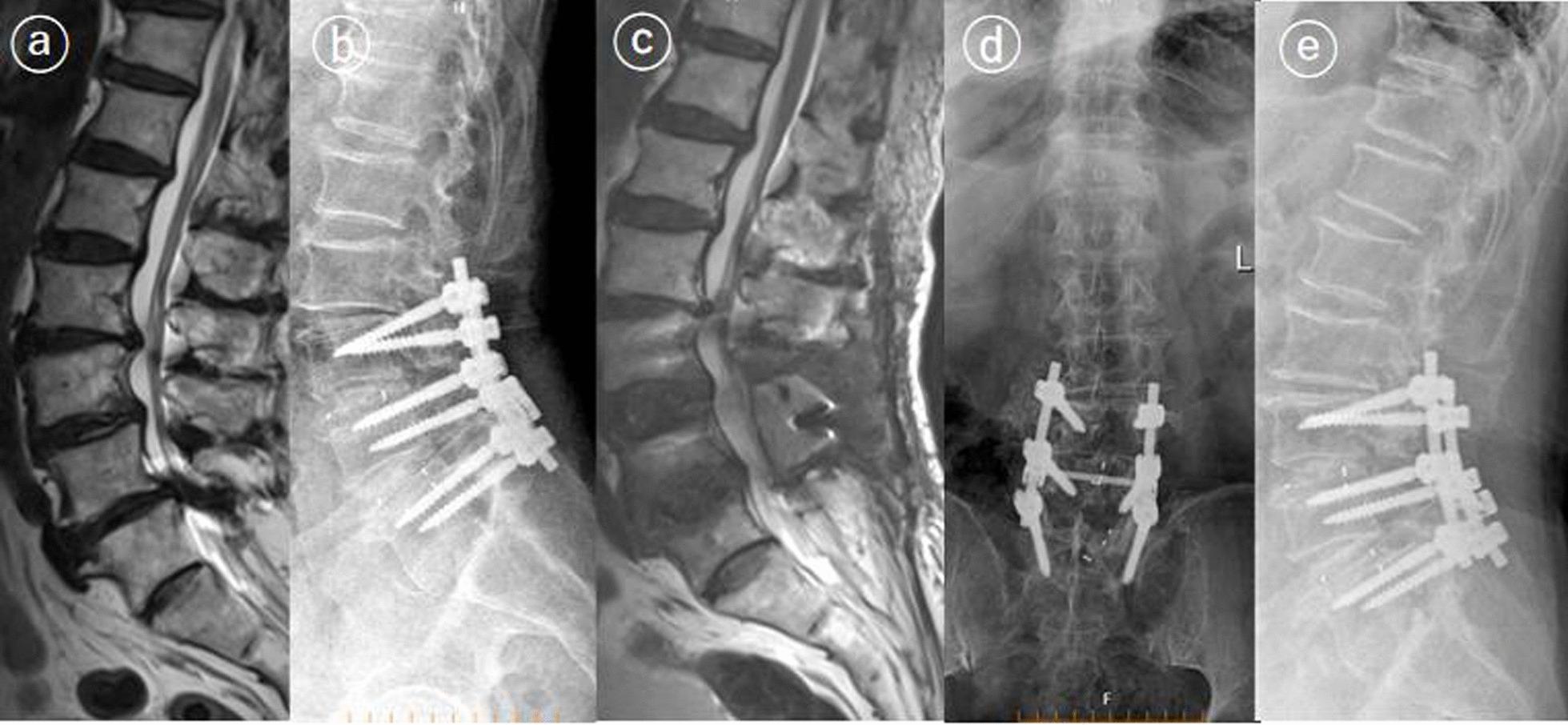
A 57-year-old patient underwent second surgery for persistent pain. The patient underwent decompression and posterior L3–L5 fusion surgery for lumbar spondylolisthesis and lumbar spinal stenosis (**a**–**c**). Preoperative leg pain was not adequately relieved after the first operation and lumbar spine MRI revealed residual stenosis at L2–L3 and L3–L4 (**c**). After adequate decompression surgery, the patient’s pain was relieved (**d**, **e**)

**Fig. 3:**
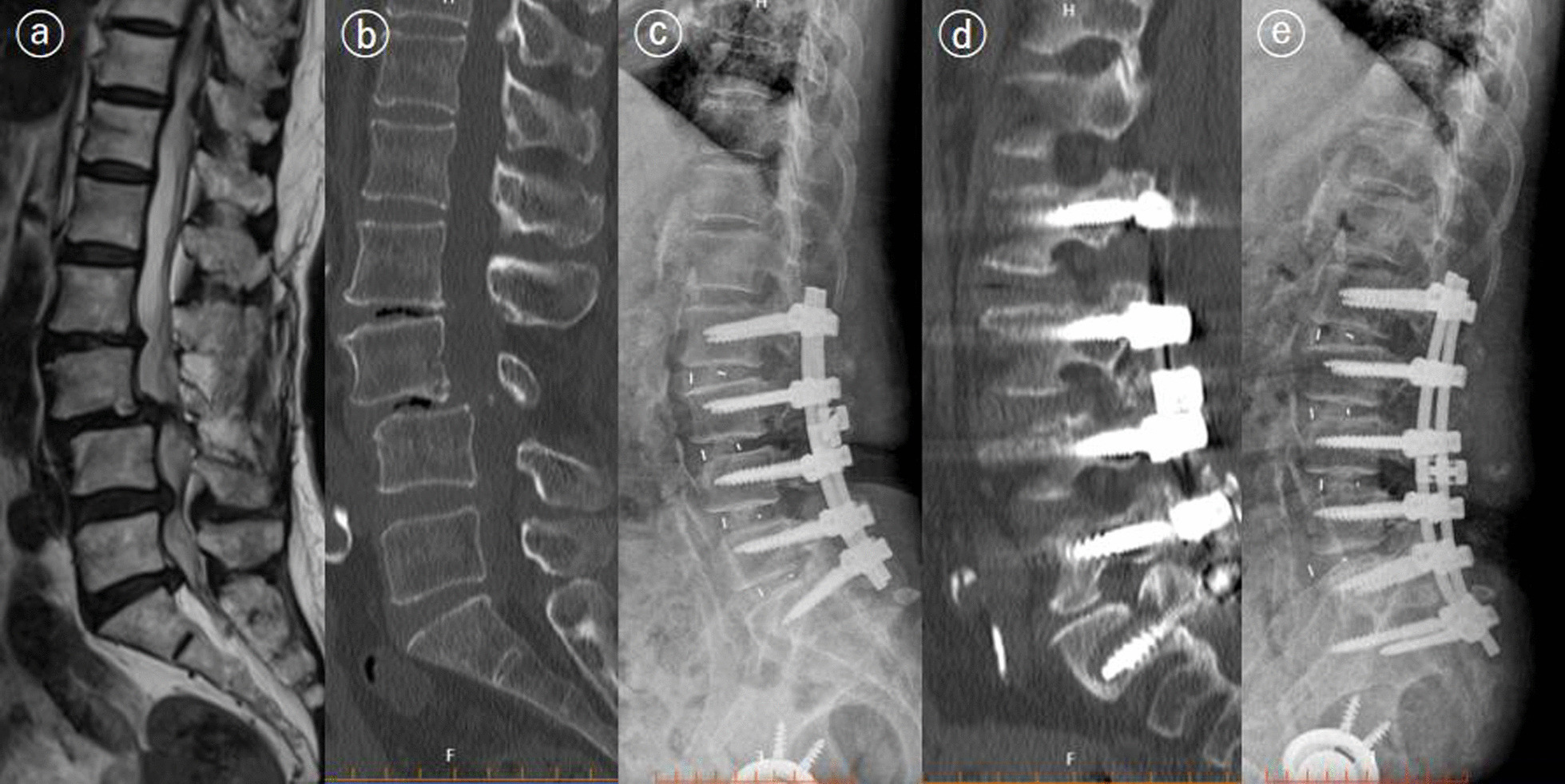
A 74-year-old patient underwent second surgery for screw misplacement. The patient underwent posterior decompression and fusion surgery for L3–L4 grade 2 spondylolisthesis and stenosis (L4–L5, L5–S1) (**a**, **b**). The postoperative X-ray was normal (**c**). The patient had moderate pain, and CT revealed bone union and screw displacement at the 1-year follow-up (**d**). The patient underwent revision surgery and achieved adequate pain control (**e**)

**Fig. 4:**
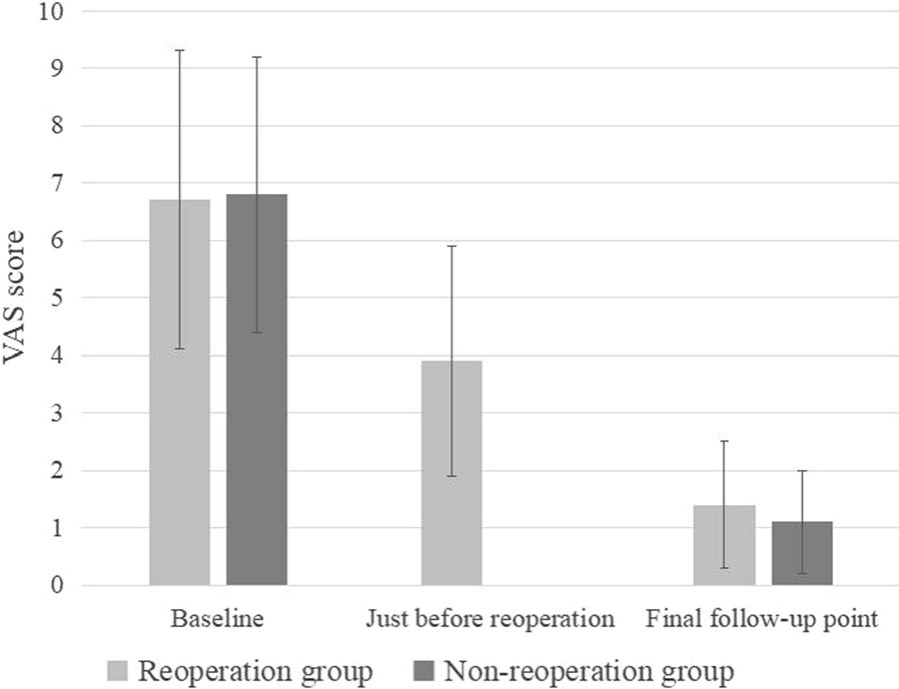
VAS score at different follow-up points in both groups

Between January 2017 and March 2020, 826 patients who underwent lumbar fusion surgery met the inclusion criteria. Three patients were excluded due to loss of contact. Two patients died of cancer 2 years after fusion surgery. Of 821 patients included in our studies, 34 patients underwent reoperation within 3 months (early reoperation), and 36 underwent reoperation 3 months or more after surgery (late reoperation). The cumulative reoperation rate was about 4.1% (95% confidence interval [CI], 3.8–4.5%) at 3 months, 6.2% (95% CI 5.9–6.5%) at 1 year and 8.2% (95% CI 8.0–8.5%) at 3 years (Fig. 1). The most common indications for early and late reoperation were SSI (32.3%) and ASD (38.9%), respectively. Hematoma, residual stenosis (Fig. 2) and instrumentation failures (Fig. 3) were also common reasons for reoperation (Table 1). The mean follow-up time for VAS scores was similar in both groups (34 ± 8.2 vs 36 ± 9.1, *p* = 0.83). The changes in VAS score at different follow-up points in the reoperation and non-reoperation groups are shown in Fig. 4. There was no significant difference in the VAS score at baseline, and both groups significantly improved the average VAS score after operation or reoperation. The VAS score was lower in the non-reoperation group than in the reoperation group at the final follow-up point (1.1 ± 0.9 vs. 1.5 ± 1.1, *p* = 0.01).

**Table 1:** The indications and rates of reoperations in early reoperation group and late reoperation group

Early reoperation (*n* = 34)	*n* (%)	Late reoperation (*n* = 36)	*n* (%)
Hematoma	8 (23.5%)	Pain recurrence	3 (8.3%)
SSI	11 (32.3%)	SSI	6 (16.7%)
Persistent pain	4 (11.7%)	ASD	14 (38.9)
ASD	3 (8.8%)	Late instrumentation failures	13 (36.1)
Early instrumentation failures	7 (20.6%)		
Cerebrospinal fluid leakage	1 (3.0%)		

SSI, surgical site infection; ASD, adjacent segment diseases

Baseline demographics, comorbidities and surgical data of patients in reoperation and non-reoperation groups are displayed in Table 2. Compared to patients who did not undergo reoperation, patients in the early reoperation group were more likely to have diabetes (44.1% vs. 24.4%, *p* = 0.01) and osteoporosis (14.7% vs. 3.9%, *p* = 0.01). The average operative time of patients in the early reoperation group was longer than patients in the non-reoperation group (247.2 ± 93.8 vs. 216.2 ± 70.1, *p* = 0.01). Patients with older age (68.1 ± 11.7 vs. 64.9 ± 11.1, *p* = 0.09) and more fused levels (38.2% vs. 24.2%, *p* = 0.06) tended to be more likely to undergo early reoperation. Compared with patients in non-reoperation group, longer operative time (243.2 ± 73.8 vs. 216.2 ± 70.1,* p* = 0.01), more EBL (596.1 ± 352.9 vs. 435.1 ± 343.1, *p* = 0.01) and fused levels (52.8% vs. 24.2%, *p* = 0.01) were observed in late reoperation group. There were no significant differences between both groups in the remaining variables (sex, BMI, primary diagnosis, other comorbidities, laboratory values and ASA). Ultimately, multivariable logistic regression analyses including age, operative time, osteoporosis, diabetes, surgical history and fused levels were conducted to identify independent risk factors for early reoperation and multivariable logistic regression analyses including weight, BMI, operative time, number of fused levels and EBL were conducted to identify independent risk factors for late reoperation.

**Table 2:** Baseline demographics, comorbidities and surgical data for reoperation group and non-reoperation groups

Variables	Non-reoperation	Reoperation			
		Early reoperation	*P*	Late reoperation	*P*
Demographic data					
Age (yr)	64.9 ± 11.1	68.1 ± 11.6	0.09	66.9 ± 9.5	0.28
Female *n* (%)	455 (60.6%)	21 (60%)	0.89	19 (52.7%)	0.35
Weight (kg)	68.7 ± 11.5	71.2 ± 11.3	0.22	73.9 ± 11.4	**0.01**
BMI (kg/m^2^)	25.9 ± 3.6	26.3 ± 3.6	0.43	27.1 ± 3.5	**0.04**
Comorbidities *n* (%)					
Cardiovascular	401 (49.6%)	10 (29.4%)	0.14	12 (33.3%)	0.35
Diabetes	183 (24.4%)	15 (44.1%)	**0.01**	7 (19.4%)	0.50
Mental disease	14 (1.7%)	1 (2.9%)	0.65	0	0.41
Digestive disease	26 (34.6%)	3 (8.8%)	0.11	3 (8.3%)	0.13
Old cerebral infarction	21 (2.9%)	2 (5.9%)	0.29	1 (2.8%)	0.90
Pulmonary diseases	16 (2.1%)	2 (5.9%)	0.15	2 (5.5%)	0.18
Osteoporosis	29 (3.9%)	5 (14.7%)	**0.01**	3 (8.3%)	0.23
Surgical history	274 (36.5%)	17 (50.0%)	0.09	15 (41.6%)	0.53
Parkinson disease	9 (1.2%)	1 (2.9%)	0.38	1 (2.8%)	0.41
Nutrition status					
Albumin (g/L)	39.5 ± 3.5	39.7 ± 4.2	0.58	40.0 ± 3.6	0.30
Prealbumin (g/L)	236.2 ± 54.0	243.4 ± 51.4	0.45	246.3 ± 55.0	0.63
Hemoglobin (g/L)	133.7 ± 15.5	133.5 ± 15.0	0.95	136.4 ± 14.5	0.24
Symptom duration (y)	6.8 ± 7.7	6.6 ± 6.7	0.99	7.7 ± 8.5	0.91
Primary diagnosis *n* (%)			0.79		0.94
DDD	184 (24.5%)	9 (26.4%)		7 (19.4%)	
Lumbar stenosis	354 (47.1%)	14 (41.2%)		18 (50.0%)	
Lumbar spondylolisthesis	213 (28.4%)	11 (32.4%)		11 (30.6%)	
Surgical data					
ASA	2.2 ± 0.5	2.2 ± 0.4	0.70	2.4 ± 0.5	0.12
Operative time (min)	216.2 ± 70.1	247.2 ± 93.8	**0.01**	243.2 ± 73.8	**0.01**
EBL (ml)	435.1 ± 343.1	501.6 ± 354.2	0.27	596.1 ± 362.9	**0.01**
Fusion level			0.06		**0.01**
1–2	569 (75.8%)	21 (61.8%)		17 (47.2%)	
3–5	182 (24.2%)	13 (38.2%)		19 (52.8%)	

Bold font indicates statistical significance

BMI, body mass index; DDD, degenerative disk disease; ASA, American Society of Anesthesiologists; and EBL, estimate blood loss

Multivariable analysis indicated that osteoporosis (odds ratio [OR] 3.6, 95% CI 1.2–10.5, *p* = 0.02) and diabetes (OR 2.1, 95% CI 1.1–4.5, *p* = 0.04) were independently associated with early reoperation. In addition, the multivariable analysis indicated that multilevel fusion (the number of fused levels > 2) (OR 2.4, 95% CI 1.0–5.4, *p* = 0.03) was independently associated with late reoperation. However, the remaining variables including age, weight, BMI, operative time and EBL were not significantly associated with reoperation (Table 3).

**Table 3:** Multivariable regression analysis for early and late reoperation

Variables	Early reoperation		Late reoperation	
	Odds ratio (95% CI)	*P*	Odds ratio (95% CI)	*P*
Age	1.0 (0.9–1.0)	0.77	–	–
Weight	–	–	0.9 (0.9–1.0)	0.12
BMI	–	–	1.0 (0.8–1.1)	0.84
Operative time	1.0 (0.9–1.0)	0.15	1.0 (0.9–1.0)	0.94
Osteoporosis	3.6 (1.2–10.5)	**0.02**	–	–
Diabetes	2.1 (1.1–4.5)	**0.04**	–	–
Surgical history	1.5 (0.3–4.3)	0.22	–	–
Fused levels > 2	1.3 (0.5–3.1)	0.51	2.4 (1.1–5.4)	**0.03**
EBL	–	–	1.0 (0.9–1.0)	0.43

Bold font indicates statistical significance

BMI, body mass index; EBL, estimated blood loss

## Discussion

Lumbar fusion surgery is a standard procedure for treating degenerative lumbar disease. Despite advances in surgical technique and implants over the past two decades, the rate of unplanned reoperation did not decrease with the increase in lumbar fusion volume [2, 6, 13]. In the present study, we found that the cumulative incidence of unplanned reoperation was about 4.1% at 3 months, 6.2% at 1 year and 8.2% at 3 years. A wide range (3.4–14.4%) of reoperation rates following fusion surgery was reported in previous publications [7, 9, 14, 15]. The rate of unplanned reoperation in our patient cohort was broadly in line with these results. The changes in cumulative reoperation rate and reason for reoperation at different times were demonstrated in prior studies [7, 9]. The present study aimed to identify the indications and risk factors for early reoperation and late operation following fusion surgery.

There are differences in reasons for reoperation between the early reoperation group and the late reoperation group. The primary reasons for the early reoperation in the present study were SSI and hematoma, followed by early instrumentation failures, persistent pain and ASD. The results of our study were similar to previous studies. Liu et al. [14] conducted a multicenter study and reported that the reasons for reoperations within 3 months after lumbar fusion surgery included wound infection (45.4%), screw misplacement (25.6%), cerebrospinal fluid leakage (13.0%), wound hematoma (8.7%) and neurologic deficit (7.2%). In another study, Durand et al. [16] found that reasons for unplanned reoperation within one month after fusion surgery were mostly infection and hematoma; the present study’s most common indication for late reoperation was ASD, consistent with a previous study [17]. A prospective cohort study by Irmola et al. [9] also reported a high incidence of ASD, and the reoperation for ASD was performed at a mean of 2.3 years after fusion surgery. We also found that SSI and pseudoarthrosis were common reasons for readmission to reoperation 3 months or more after surgery. By analyzing the indication and timing of reoperation, this study demonstrated that the surgeon should be focused on different issues according to the length of time after fusion surgery.

Postoperative pain in the lower back or leg is a major complaint among patients receiving reoperation. We found that lumbar fusion surgery provided efficient pain relief in patients with the degenerative lumbar disease, and most patients experienced a significant reduction in VAS scores after fusion surgery. However, some patients required a second procedure to achieve adequate pain relief. We found that the VAS scores of these patients remained higher than those who did not undergo the second procedure at the final follow-up, which was consistent with the results of previous studies [7, 18]. In a retrospective study of 309 patients, Montenegro et al. [19] found that up to 23% of patients had a declined functional status 6 months after reoperation. The underlying reason for this is unclear; however, more extended hospital stays and higher costs may lead to lower satisfaction and confidence in the operation [20, 21].

By multivariable analysis, we found that osteoporosis and diabetes were risk factors for early reoperation; however, the remaining variables did not affect the rate of early reoperation after lumbar fusion. Osteoporosis is characterized by a reduction of bone mineral density and is diagnosed based on X-ray, computed tomography and dual-energy X-ray absorptiometry. Patients with osteoporosis risk implant displacement including screw loosening, cage sinking and fractures [22, 23]. Khalid et al. [24] reported that osteoporosis was independently associated with pseudoarthrosis and revision surgery in adult patients undergoing single-level lumbar fusion. Previous studies have also found that vertebral osteoporosis is a risk factor for adjacent vertebral fractures and for proximal junctional kyphosis after multilevel fusion [25, 26]. For patients diagnosed with osteoporosis, the reduced number of manipulations and the use of bone cement may reduce the incidence of screw loosening and adjacent vertebral fractures [27]. Diabetes is associated with poor wound healing. Golinvaux et al. [28] reported that patients with diabetes had a higher incidence of postoperative wound infection than other patients. Kim et al. [29] performed a multivariate regression analysis and found that diabetes was an independent risk factor for unplanned reoperation after fusion surgery, which was consistent with our findings. Insufficient local blood supply, poor immunity and neurological damage increase the risk of surgical wound infection in patients with diabetes [30]. Rathmann et al. [31] found that diabetes was associated with a higher rate of fractures. Early reoperation is usually performed for debridement and depression in patients with long-term wound infection and residual stenosis. We did not find any other remaining variables associated with early reoperation, including age, other comorbidities and ASA score. In a retrospective study of 22,151 patients, Durand et al. [16] found that obesity, ASA, disseminated cancer, weight loss and multilevel fusion were identified as significant risk factors for reoperation within 30 days following elective posterior lumbar spinal fusion. Differences in the study population may significantly impact on the results of different studies. While the purpose of the different fusion techniques is similar, their surgical approach and extension of trauma vary [32]. In the present study, we only included patients who underwent open TLIF surgery including long- and short-segment fusion.

We found that only the number of fused levels > 2 was an independent risk factor for late reoperation. This result is similar to Durand’s study, which reported that long-segment fusion was independently associated with reoperation during long-term follow-up [16]. In long-segment fusions, surgical procedures destroy more paraspinal muscles and alter spinopelvic sagittal parameters. Compared with short-segment fusion, long-segment fusion has a higher risk of adjacent segment degeneration [17]. Furthermore, previous studies have found that the number of fused levels > 2 was an independent risk factor for revision surgery due to screw loosening and rod breakage [33]. ASD and late implant displacement mostly cause persistent lower back pain and can only be detected by imaging examination. Persistent lower back pain should be given more attention in patients undergoing long-segment fusion.

Few published studies have reported differences in risk factors between early and late reoperation to the best of our knowledge. In the present study, we compared characteristics, laboratory tests, primary diagnosis and surgery-related variables in the reoperation and non-reoperation groups in the same patient cohort and found that diabetes and osteoporosis were independently associated with early reoperation and long-segment fusion was independently associated with late reoperation. Our study demonstrates the association between the risk factors of reoperation and the duration of follow-up. In future studies, follow-up time should be considered an independent variable when evaluating outcomes of patients undergoing lumbar fusion surgery.

There are some limitations to this study. First, the differences in surgical indications and techniques among hospitals significantly impact reoperation rates; nevertheless, we could not verify this due to the nature of single-center retrospective studies. Second, patients’ radiographic data, such as sagittal parameters, were not recorded; therefore, we could not compare the degree of lumbar spondylolisthesis and the grade of disk degeneration between groups. Third, although the same surgical team performed the surgery, each individual's specific surgical procedure, including decompression of the segment and lateral area, is challenging to analyze. Moreover, due to the data loss on the Oswestry Disability Index in some patients, we did not compare the functional status of the two groups. Further prospective multicenter studies should include patient imaging data and expand the sample size to draw more reliable conclusions.

## Conclusions

The cumulative incidence of unplanned reoperation was about 4.1% at 3 months, 6.2% at 1 year and 8.2% at 3 years after lumbar fusion surgery. Although the patient's lower back pain was significantly improved after the reoperation, the VAS score was lower in the non-reoperation group than in the reoperation group at the final follow-up point. The most common reasons for early reoperation and late operation were SSI and ASD, respectively. Multivariate analysis revealed that osteoporosis and diabetes were independently associated with early reoperation, whereas multilevel fusion was independently associated with late reoperation. Our results suggested that surgeons should pay more attention to these patients and future studies should consider the effects of follow-up periods on results.

## Data Availability

The underlying data supporting the results of this study could be obtained by contacting the corresponding author.

## Abbreviations

TLIF: Transforaminal Lumbar Interbody Fusion
BMI: Body Mass Index
ASA: American Society of Anesthesiologists
EBL: Estimated Blood Loss
VAS: Visual Analog Scale
SSI: Surgical Site Infection
ASD: Adjacent Segment Diseases
